# ‘We did everything by phone’: a qualitative study of mothers' experience of smartphone-aided screening of cerebral palsy in Kathmandu, Nepal

**DOI:** 10.1186/s12887-024-04829-5

**Published:** 2024-05-22

**Authors:** Antti J. Kukka, Pratiksha Bhattarai, Heléne E. K. Sundelin, Rejina Gurung, Nick J. W. Brown, Helena Litorp, Anna Axelin, Ashish KC

**Affiliations:** 1 Department of Women’s and Children’s Health, Uppsala University, Uppsala, SE-751 85 Sweden; 2 Department of Pediatrics, Gävle Regional Hospital, Gävle, Region Gävleborg Sweden; 3 Golden Community, Lalitpur, Nepal; 4 Department of Biomedical and Clinical Sciences, Division of Children’s and Women´S Health, Linköping University, Linköping, Sweden; 5 Department of Women’s and Children’s Health, Neuropediatric Unit, Karolinska University Hospital, KarolinskaInstitutet, Stockholm, Sweden; 6 Department of Global Public Health, Karolinska Institutet, Stockholm, Sweden; 7 Department of Nursing Science, University of Turku, Turku, Finland; 8 School of Public Health and Community Medicine, University of Gothenburg, Gothenburg, Sweden

**Keywords:** Cerebral palsy, Qualitative research, General movements assessment, Neonatal follow-up, Low- and middle-income countries, Telemedicine

## Abstract

**Background:**

International guidelines recommend early intervention to all children at risk of cerebral palsy, but targeted screening programs are often lacking in low- and middle-income settings with the highest burden of disease. Smartphone applications have the potential to improve access to early diagnostics by empowering parents to film their children at home followed by centralized evaluation of videos with General Movements Assessment. We explored mothers’ perceptions about participating in a smartphone aided cerebral palsy screening program in Kathmandu, Nepal.

**Methods:**

This is an explorative qualitative study that used focus group discussions (*n* = 2) and individual interviews (*n* = 4) with mothers of term-born infants surviving birth asphyxia or neonatal seizures. Parents used the NeuroMotion™ smartphone app to film their children at home and the videos were analysed using Precthl’s General Movements Assessment. Sekhon et al.’s framework on the acceptability of health care interventions guided the design of the group discussions and interviews, and the deductive qualitative content analysis.

**Results:**

Mothers were interested in engaging with the programme and expressed hope it would benefit their children. Most felt using the app was intuitive. They were, however, unclear about the way the analysis was performed. Support from the research team was often needed to overcome an initial lack of self-confidence in using the technology, and to reduce anxiety related to the follow-up. The intervention was overall perceived as recommendable but should be supplemented by a face-to-face consultation.

**Conclusion:**

Smartphone aided remote screening of cerebral palsy is acceptable in a lower middle-income population but requires additional technical support.

**Supplementary Information:**

The online version contains supplementary material available at 10.1186/s12887-024-04829-5.

## Background

Cerebral palsy (CP) is a disorder of movement, tone and posture resulting from nonprogressive damage to the developing brain [1]. It is the most common form of motor disability in childhood and is often associated with other developmental problems [1]. International guidelines recommend early intervention for children at high risk of CP with the aim of improving functional outcomes by goal oriented motor training and enhancing parental capacity for attachment [2].

The majority of children affected by CP live in low- and middle-income countries (LMICs), [3] where access to early diagnostics is limited [4]. General Movements Assessment (GMA) is a free non-invasive tool for identifying infants at high risk of CP, [5] but requirement for training has limited its use in LMICs [6]. Studies conducted mainly in high-income countries show that GMA can predict CP with around 90% sensitivity and specificity at 3 months’ age and thereby enable targeted early intervention [7, 8].

Telehealth has the potential to improve access to diagnostics across the globe [9]. In high income countries, smartphone applications have already empowered parents to contribute to the follow-up of their children using remote GMA [10–12]. Remote GMA could overcome the access barrier to early CP diagnosis and engaging parents in the follow-up can spare limited healthcare resources in LMICs [13].

We conducted a pilot study testing the feasibility of smartphone-aided remote GMA for identifying children at high risk of CP in Kathmandu, Nepal. The NeuroMotion™ app developed by Linköping University, Sweden, [10] was translated into Nepali and provided to parents of infants at risk of CP due to birth asphyxia or neonatal seizures [14].

Acceptability is one of the key areas of assessing digital health interventions for health systems strengthening according to the World Health Organization Guidelines [15]. Davis’s Technology Acceptance Model postulates that acceptability is critical in predicting real-world usage of technological interventions [16]. We therefore aimed to explore mothers’ perceptions of participating in smartphone-aided remote developmental follow-up of their infants in Nepal. The results of the study will guide the overall feasibility assessment and potential future scale-up of the screening program. Pragmatic philosophy guided the inquiry as we wanted to learn how to modify the intervention based on the study findings.

## Methods

Standards for Reporting Qualitative Research [17] and the Consolidated Criteria for Reporting Qualitative Research (COREQ) guidelines were used for writing the report [18].

### Design

This is an explorative qualitative study using focus group discussions (FGD) and individual in-depth interviews (IDI) of mothers participating in the smartphone aided GMA follow-up of their children.

### Theoretical framework

**Fig. 1:**
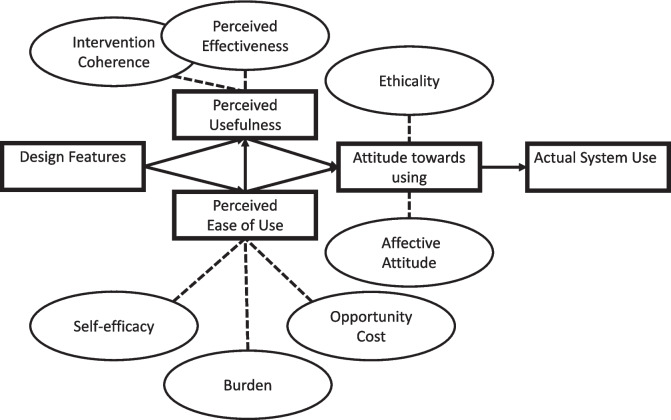
Theoretical framework predicting the use of a technological intervention. Adapted from Technology Acceptance Model by Davis [15] and Acceptability of Healthcare Interventions by Sekhon et al [18]

The Technology Acceptance Model by Davis suggests that actual system usage is determined by the overall attitude of the user towards a given system [16]. This attitude stems from two cognitive responses to the design features of the intervention: perceived ease of use and usefulness [16]. Sekhon et al. give seven constructs of acceptability that can be used to further elaborate the Technological Acceptance Model (Fig. 1) [16, 19]. They define acceptability as ‘a multi-faceted construct that reflects the extent to which people delivering or receiving a healthcare intervention consider it to be appropriate, based on anticipated or experienced cognitive and emotional responses to the intervention’ [19]. They also suggest that acceptability should be examined prospectively, concurrently, and retrospectively in relation to the intervention use.

### Study setting

Nepal is a multi-ethnic country situated between China and India along the Himalayan Mountain range. Over the last two decades, the nation has experienced economic growth with the World Bank classification changing from low to lower-middle income status in 2020. In 2022, 80% of women and 92% of men owned a mobile phone [20]. Several previous studies have explored the use of telemedicine to bridge the access barriers to healthcare services caused by the geographical and staffing challenges in the country [21].

This study took place in Kathmandu Valley, which consists of three intergrown cities one of which is the capital of the country. Kathmandu is the economic powerhouse of Nepal with the highest levels of education and access to communication technologies [20]. Institutional deliveries have become the norm with above 80% of urban women delivering in a health facility in 2022 [20].

The epidemiology of CP in Nepal is poorly known as most available studies are facility based and only one population-based provincial register exists [22]. In comparison to high-income countries, a larger proportion of children with CP are born term after birth asphyxia or suffer from post-natal complications [23]. Care for children affected by CP is provided by non-governmental organizations working together with the Ministry of Health utilizing the World Health Organization Community Based Rehabilitation model [24].

Follow-up of the children and the remote GMA assessment are described in a separate paper [14]. In short, recruitment of children took place at Paropakar Maternity and Women’s Hospital (PMWH), the largest public tertiary maternity hospital in Kathmandu. Parents to 31 infants surviving birth asphyxia or neonatal seizures were instructed to install the NeuroMotion™ app into their smartphones. At three months’ age, the parents received a notification from the app requesting that they send a film of their child’s spontaneous movements, which were analysed by the research team using Prechtl’s qualitative GMA with results reported back to parents within 2 weeks of filming. Children with absent age-typical fidgety movements indicating a high risk of CP were referred for paediatrician’s evaluation at the PMWH and recommended early intervention at a non-governmental rehabilitation organization Self-help Group for Cerebral Palsy.

### Study population

A purposive sample of 12 mothers participating in the smartphone aided follow-up of their child were recruited for two FGDs with four participants each and four IDIs. Research assistants involved in the recruitment of the children and their follow-up helped to select information rich cases, who based on their previous experience with the families, were thought to have varied opinions about the program.

The study participants comprised of mothers from diverse socioeconomic backgrounds, representing a range of educational levels from some years of primary school to completion of a university degree. Various castes and ethnicities, including both relatively advantaged and disadvantaged groups, were included in the study. All participating families had access to a smartphone, although not all mothers owned one personally. Specific demographic details, such as age, place of residence, occupation, parity, and ethnicity, were not reported to maintain participant confidentiality. Additionally, participants' prior knowledge about CP was not probed to avoid potential bias in responses. A previous ethnographic study conducted in Nepal suggests that the condition is generally recognized and explained by theories ranging from the biomedical to ayurvedic and to spiritual [25].

We chose to have FGDs with parents whose children had successfully been filmed and judged to have normal fidgety movements to elucidate a variety of opinions about the follow-up program as the topic was not considered to be too sensitive for a group discussion. IDIs were chosen when the child had absent fidgety movements indicating a high risk of CP (*n* = 2) and when filming was not completed successfully (*n* = 2) to ensure the privacy of the participants.

Potential participants were approached by telephone by a research assistant after providing the results of the GMA analysis. Children who were not successfully filmed during the study were offered additional neurological assessment at one year of age and their mothers’ willingness to participate was assessed at the same time. No parent refused to join, but one mother in each of the scheduled FGDs did not show up to the appointment. Reasons for drop-outs were not inquired. Informed consent was collected both in writing and orally for audio recording of the interviews and the following data-analysis and publication.

### Data collection

Semi-structured interview frameworks were developed separately for the FGDs and IDIs based on Sekhon et al.’s acceptability framework (Fig. 1) [19]. The guide was first piloted in five interviews (one single mother, two single fathers and two couples) who had not yet left the delivery hospital with focus on the parents’ expectations about the follow-up. We found that it was difficult for the participants to discuss the program acceptability prospectively. These interviews were short, approximately 15 min each, and superficial, and were therefore only used for adjusting the interview guides. Minor edits to the guide were also permitted thereafter to ensure that all the relevant topics were covered (Supplementary material: Interview Guide). Also based on the pilot experience, only mothers were invited to join the IDIs and FGDs.

A private location was selected for the group discussions and interviews at the PMWH. During the sessions, a female relative of the mothers took care of the infants. During one of the two FGDs the babies and the relatives were present in the room while in the other FGD and all the IDIs they waited outside. Both the group discussions and individual interviews started with an open question about the current health of the children participating in the follow-up before moving on to discuss the mothers’ attitudes and experiences before, during and after the use of the app for filming their child. The FGDs lasted 40 to 60 min each and all mothers participated actively. The IDIs were 20 to30 minutes each. Data analysis was performed concurrently with data collection and the exact number of interviews was determined by the point of data redundancy, which was achieved after the first IDI conducted with a mother whose infant had not been successfully filmed. No repeat interviews were done.

The FGDs and IDIs were conducted by a Nepalese female researcher with a background in nursing and child development and previous experience in qualitative research (PB). The interviews were observed by two Nepalese female public health students. One student was present at each session with responsibility for note keeping and both transcribed the audio recorded data verbatim and translated it from Nepali to English. Proof reading and finalization of the transcripts were undertaken by the interviewer. The finalized transcripts were not returned to the interviewees for review. Additional field notes were gathered throughout the data collection period and informal discussions with the research assistants in charge of patient recruitment and follow-up were held to better understand some of the answers given by the mothers during the interviews. All data were stored on a password protected hard drive.

### Data analysis

Qualitative content analysis presented by Patton was used to analyse the data [26]. A deductive approach building on seven constructs of acceptability defined by Sekhon et al. guided the data analysis (Fig. 1) [19].

One researcher (AK) coded the data. Manual data analysis was conducted, and findings were discussed in a multi-professional team consisting of both Nepali (PB) and European (AK and AA) researchers. Transcripts and notes were read repeatedly, and comments were made to margins to identify meaning bearing units. These were tagged with a defining code. Codes were grouped into categories consisting of similar content [26]. Sekhon et al.’s seven constructs of acceptability were used as primary themes, but the codes and categories were then examined and refined until the categories became mutually exclusive. Sub-categories were used to aid in reaching convergence [26].

Constructs ‘Burden’ and ‘Opportunity Costs’ included in Sekhon et al.’s framework were merged as it was not possible to sufficiently differentiate the respondents’ answers between these themes. An example of a partial coding tree for two of the categories is provided in the Supplementary Figure to exemplify the analytical process.

### Reflexivity

The author team comes from academic and clinical background from Nepal and Northern Europe differing from that of many mothers interviewed, who sometimes only had elementary level education. Our training is based on the Western biomedical concept of explaining illness and disability whereas a plurality of interpretations are encountered in Nepal [25]. Parents of children with disability commonly seek care from providers of different epistemological backgrounds in Nepal [27]. The goal of the study was not to explore what disability means to participating families but to find out how they perceive the follow-up method. As such, cultural differences are likely to be less problematic. The fact that part of the research team comes from outside the caste system dictating social roles in Nepal could also be seen as beneficial for the interpretation of findings. Gender of the interviewers and interviewees were matched.

### Ethics

Participating in the FGDs and IDIs might have caused anxiety in mothers who had been through a difficult delivery and thereafter been told that their child might develop disability. At the same time, it allowed parents to voice their view of the ethicality of the whole screening program. We reserved time for mothers’ questions about the follow-up after each meeting and tried to ensure further referral of any medical needs of the children involved in the study. Parents who requested physical examination of their baby were provided the opportunity to meet a paediatrician from PMWH at the hospital’s out-patient department.

## Results

The findings of the analysis are presented in Table 1. Sekhon’s acceptability framework formed the overarching themes under which two to four categories for each theme were placed. All interviews were conducted after the parents had used the app, but we considered each analytical category in relation to time from app use.

**Table 1:** Acceptability of smartphone-aided screening of cerebral palsy in Kathmandu, Nepal

Themes	Categories			
*Time:*	*Prospective to app us*	*Concurrent to app use*		*Retrospective to app use*
Affective attitude	Curious mothers - Initially positive - Nervous	Anxiety during follow-up		Relief, distress or disappointment
Perceived effectiveness	Benefit to the child - Knowledge - Help - Counselling	Direct contact with doctors	Certainty of face-to-face meeting	A recommendable intervention
Ethicality	Dedicated research team	Slow remote assessment		Unmet expectations
Intervention coherence		Limited understanding of the intervention - Short videos - Trust in doctors		A mother knows best?
Self-efficacy		Easy and intuitive app	Lack of self-confidence	Technical hinders
Burden and Opportunity cost		Busy life		Easier with a familiar app

### Affective attitude

#### Curious mothers

##### Initially positive

The initial feelings of the participants towards the follow-up had mostly been positive. Many expressed that they were happy that such a program was offered for their children and were satisfied with the information received during the consent taking. Some were fascinated by the idea of remote follow-up as a novel intervention that had not been offered by the hospital before.

IDI Absent GMA 1: *‘At that time, I completely agreed with her* (the research assistant)*. When she said that the baby will be followed- up, I really like that idea.’*

##### Nervous

Many mothers had also been nervous about joining the program. They all had gone through some form of neonatal complications with related anxiety. Some had previous experience of similar deliveries when no remote follow-up was in place and thus expressed initial scepticism towards the study. They voiced their concern for misuse of videos and had on few occasions been advised by family members or other parents in the ward against joining the study. This scepticism might have been partly explained by the fact that not all mothers were present during the initial consent taking and only received information about their child’s enrolment later from their husbands.

IDI Absent GMA 1: ‘*There are so many so many things we hear these days. People say these videos are for research, but they use it for some other purposes.’*

#### Anxiety during follow-up

Worry and anxiety were present during the whole course of the follow-up. Mothers restlessly waited for time of filming to come. At least for some, participating in the follow-up itself increased anxiety. Others, however, denied additional worry when asked directly.

IDI Failed Film 2*: ‘As we were continuously followed-up *via* phone, and were shown greater concern, I was really stressed.’*

#### Relieved, distressed or disappointed

For those mothers whose children were determined to have normal fidgety movements at 3 months’ age, worry soon dissipated. These parents were happy that they had joined the study and relieved to learn that their child was doing well despite the early life complications.

FGD 2, respondent 2*: ‘Now, you have informed me that my baby is fine and does not have any abnormality, I feel delighted.’*

However, in cases where the assessment was abnormal, anxiety continued until the time of the interview. Mothers who had for some reason failed to upload a film for evaluation were disappointed and felt let down by the research team.

IDI Failed Film 2: *‘Till now, I have that* (worry) *in my heart.’*

### Perceived effectiveness

#### Benefit to the child

The primary motivation for participating in the study was perceived general benefit to the child. Mothers were hoping to gain *knowledge* about their infants’ health and development. They also expressed hope that through enrolment in the study they would be *help*ed in case difficulties arose within these domains. The research assistants in charge of patient recruitment and follow-up were sometimes seen as a type of a *counsellor* who would help to navigate the health care system in case treatment was needed.

FGD2, Respondent 1: *‘We all joined because we were very hopeful that you will guide us in a proper direction to either treat the baby or to take care of the baby.’*

#### Direct contact with doctors

Many mothers viewed the program as an opportunity for direct access to doctors in the delivery hospital. Joining the study was perceived to facilitate contact with the paediatricians who knew about the initial challenges of their children and had access to the GMA results. Understanding the separation between the research study and routine care was not always easy for the parents.

IDI Absent GMA 1: *‘I thought I will need to treat my baby, so why not to join this program which would benefit me to easily contact the doctors present here.’*

#### Certainty of the face-to-face meeting

The effectiveness of the remote video assessment was often perceived as less complete than a physical evaluation. A physical appointment enables two-way communication that was lacking from the remote check-up, which led some mothers to voice their preference for a traditional visit to a doctor. In particularly the mothers whose children had absent fidgety movements and those who had not been able to film according to the plan appreciated the opportunity for a confirming physical evaluation.

IDI Absent GMA 2: *‘We can talk normally face to face and it will be certain.’*

#### A recommendable intervention

Having completed the pilot study, a few mothers spontaneously voiced their recommendation for the remote follow-up to be offered to other children and in other hospitals too. No participant openly opposed the idea of continuing remote assessment despite facing some challenges during the study.

FGD2, Respondent 3: *‘We hope now other babies also gets* (SIC) *benefit from it.’*

### Ethicality

#### Dedicated research team

A major factor for the overall acceptability of the follow-up was the interaction between research assistants and the participants. The fact that the research assistants showed interest in the children and the parents was greatly appreciated. They also went the extra mile trying to ensure that everyone would have the opportunity to be filmed by sometimes agreeing to meet those struggling with the app either at home or at the delivery hospital. The support experienced by most participants was in stark contrast to the disappointment felt by those who for some reason had failed to film successfully.

FGD2, Respondent 2: *‘As I got the opportunity to have a face-to-face talk with you, this has made me more confident regarding my baby’s condition’.*

Had the mothers been initially sceptical about joining the study, many expressed that the opportunity to ask questions during follow-up helped to reduce the worry. Sometimes, however, family dynamics hindered this communication with only fathers having access to the mobile phone and mothers receiving information only through their husbands.

FGD2, Respondent 2: *‘I wanted to talk with you directly but my husband thinks I will worry and take a lot of tension. So, he would never give my number to you.’*

#### Slow remote assessment

Many, but not all, parents expressed that the waiting time of up to two weeks between filming and receiving results was excruciating. This was the period of peak anxiety and a major drawback of remote assessment in comparison to direct face-to-face examination by a physician. Mothers recalled feeling afraid of disability or disease in their child and some had made plans for consulting doctors in case the report was abnormal. A minority of parents were seemingly more relaxed reporting that the waiting time did not feel very long and that feelings of curiosity rather than anxiety dominated the period.

FGD2, Respondent 2:*’I would repeatedly ask my husband if there was any message from the hospital. My husband was frustrated and would ask me how many time do you repeat and ask the same question again and again.’*

FGD2, Respondent 3: *‘I got to know about the result after 2–3 days of sending the video. I should consider it a very reasonable time.’*

#### Unmet expectations

There were also some unmet expectations such as a wish to learn more about the analytical process involved in the GMA or a request for a formal written report after completing the child’s assessment.

IDI Failed Film 2: *‘I wanted to know why this happened to my baby, what happened to my baby during operation?’.*

### Intervention coherence

#### Limited understanding of the intervention

##### Short video

Many mothers who had participated in the follow-up seemed to have vague understanding about the nature of the remote assessment done to their child. A commonly occurring concern was ‘*How would you analyse such a small video of only 2 to 3 min?*’ This was particularly prominent if the child had not been at their perceived best during the period of filming. Many parents seemed to think that the doctors would be interested in the gross movements of the child rather than the fidgety movements occurring continuously during awake state. Some would even engage in stimulating their child to elicit gross motor movements which, although understandable, was counter to the instructions for GMA.

FGD2, Respondent 1: *‘He did not had* (SIC) *much movements. Once I would* (turn) *off the video then, the baby would move his hands and feet. So I was really tensed* (SIC)*.’*

##### Trust in doctors

Other mothers, however, expressed their trust in doctors being able to assess even short videos and seeing things that parents themselves might not have noticed. It was further pointed out that the child might not be at one’s best during a physical visit either and with remote assessment several videos could be sent.

FGD2, Respondent 3: *‘I consoled myself thinking, videos will be assessed by doctors, so they will understand the 3 min video very well.’*

A couple of participants had for some reason understood that the videos could only be uploaded during office hours, which limited their ability to participate.

#### A mother knows best?

Many mothers expressed a certain gut feeling about their child’s good health despite initial worry after the delivery. Some issues arose when the parents’ perception about their child differed from the assessment and led the parents to question the findings. The potential fallacy of trusting one’s gut feeling more than experts’ evaluation was, however, also perceived as a reason for continuation of the follow-up program.

IDI Failed Film 1: *‘Because of this lack of awareness among parents, this program is indispensable not only in this hospital but also in other hospitals as well.’*

### Self-efficacy

#### Easy and intuitive app

Using the NeuroMotion™ app was generally perceived as easy by most mothers. They expressed that following the instructions was simple and that uploading videos went smoothly. The process of learning how to use a new intervention caused excitement and the ability to participate in the child’s follow-up was a positive experience for the family.

IDI Absent GMA 1: *‘This app is easily understood. Nowadays most of the women are educated, so yes, most of them could follow the instructions.’*

#### Lack of self-confidence

While the NeuroMotion™ app is designed to automatically send a notification to the parents’ phone at the time of filming, most of the mothers needed additional confirmation call from the research assistant to engage in filming. Her role in empowering parents in filming was crucial for the overall success of the program.

Some mothers received help from their relatives or the research team to complete the filming. Lack of clear confirmation from the app that a film was successfully uploaded was a cause of complaint from participants.

FGD1, Respondent 1: *‘There was a message and I thought maybe it was time to send the video. But the sister* (research assistant) *called after some days, so I was sure to film the baby.’*

#### Technical hinderances

Sometimes technical glitches reduced the self-efficacy of parents and caused frustration. A few mothers did not receive the intended notifications and on some occasions the videos filmed did not automatically upload as intended. Help from the research assistant was needed in such cases.

IDI Absent GMA 2: *‘…but I did not receive any notification. The sister* (research assistant) *had mentioned that notification would appear, but I did not received* (SIC) *notification during the first film.’*

### Burden and opportunity costs

#### Busy life

The interviewed mothers were in two camps regarding how parent-friendly the follow-up program felt. A common view was that the remote assessment saves time in comparison to queuing for a physical assessment in the hospital, although particularly those living close to the hospital also voiced their preference for the hypothetical physical check-up. Answering the follow-up phone calls and engaging in filming was also a burden but was generally considered acceptable and a sign of engagement from the part of the research assistants.

FGD2, Respondent 1: *‘We did everything by phone, it was very helpful and convenient.’*

#### Easier with a familiar app

A few parents questioned the trouble of learning the use of a new application instead of using something they already were familiar with like Facebook, and would have preferred sending videos via these channels instead.

FGD1, Respondent 3:*’You have brought this rule* (of using the NeuroMotion™ app), *but it would be easy to send the video from Imo *(a type of a messaging app) *or* (Facebook) *messenger only.’*

## Discussion

This qualitative study exploring mothers’ perceptions of smartphone-aided remote screening for CP in Nepal found that while the NeuroMotion™ app was often easy to use and the idea of remote follow-up was well received, support is needed to enable successful filming and reducing parental stress.

The Technology Acceptance Model suggests that the use of information systems is predicted by the interplay of design features and users’ attitudes towards the intervention [16]. Only half of the children recruited into this follow-up study were successfully filmed despite the overall positive affective attitude and acceptable ethicality [14]. The relatively low success rate might be explained by the fact that some of the design features of the app like automatic notifications were not as functional in Nepal as in our previous experience in Sweden [10].

Minimizing technical glitches, and improving parental self-confidence and knowledge about the screening intervention by providing hands-on training to both parents at the hospital followed by clearer feedback about the timing of filming and success or failure of upload are some of the suggestions made by the interviewed mothers that could improve the outcome of filming in the future. It is necessary to allocate resources for counselling the parents involved in the follow-up to reduce their anxiety. Our findings are partly contradictory to a recent Danish study, which found that smartphone-aided remote GMA increased parents’ sense of control during developmental follow-up [28]. Some participants in Nepal also questioned the need to learn a separate app, and a recent study from Australia found that even fairly simple instructions can help parents to correctly film their child at home using a smartphone camera and a messaging app [13]. A small pilot from India suggests that this can also be done in middle-income settings [29].

Intensive efforts are on-going to automatize GMA with the help of machine learning, [30] which could lead to a revolution in access to early CP diagnosis. Larger studies are, however, needed to confirm the validity of the method in LMIC settings [31]. Our findings suggest that scaling up GMA screening with the help of remote assessment from home requires considerable resources in LMICs and alternative means of filming such as engaging community health workers in the task could be explored. The period between sending videos and receiving the results should be minimized to reduce parental stress, and a possibility for direct face-to-face consultation should be provided for all parents. Similar desire for face-to-face assessment was also voiced by mothers participating in neurodevelopmental follow-up of their infants in England [32]. An opportunity to ask questions about their child’s health and development improves parental trust in the screening results, and provides an opportunity to screen for other adverse neurodevelopmental outcomes than CP for which GMA has lower sensitivity for [33]. Lastly, suitable early intervention for children identified to be at increased risk of CP should be available [2].

## Limitations

The acceptability framework by Sekhon et al. [19] that guided the development of our interview guide and analysis has an individualistic background and unclear transferability to Nepal, where communal decision making is common. Our findings highlight the importance of informing both parents about the follow-up already during recruitment and allowing both a chance to ask questions during the follow-up to avoid deepening the gender digital divide [34].

We interviewed mothers who had not succeeded in filming their child but were not able to reach out for those who refused to enrol in the study or were lost during the follow-up. The findings therefore represent opinions of parents who were sufficiently interested in the intervention to at least try to film their child at home. The overall transferability of the results was further limited by the pilot nature of the study and enrolment of only parents residing within the Kathmandu valley. The individual interviews were short and, due to drop-outs, the focus group discussions were conducted with only three participants with variable backgrounds, which might have reduced the depth and variety of opinions voiced. Social desirability bias might have also led to participants expressing a favourable opinion of the intervention as the interviewer came from the same organization that conducted the follow-up. Based on our pilot interviews, we only recruited mothers as the primary caregivers and the findings might have been different had fathers also participated.

## Conclusion

Remote screening of CP using the NeuroMotion™ app is acceptable to mothers in Nepal, but successful follow-up requires additional support to reduce parental anxiety and to improve their self-efficacy. The study was limited by small amount of data and findings might not be transferable beyond the urban lower middle-income setting.

### Supplementary Information

Supplementary Material 1.

Supplementary Material 2.

## Data Availability

The datasets generated and/or analysed during the current study are not publicly available due privacy of the interviewees but are available from the corresponding author on reasonable request.

## Abbreviations

CP: Cerebral palsy
FGD: Focus group discussion
GMA: General Movements Assessment
IDI: In-depth interview
PMWH: Paropakar Maternity and Women’s Hospital
