# Supplementary figures and images for: ‘We did everything by phone’: a qualitative study of mothers' experience of smartphone-aided screening of cerebral palsy in Kathmandu, Nepal
Source: BMC Pediatr. 2024 May 22;24:357. doi: 10.1186/s12887-024-04829-5 (PMC11110401; doi:10.1186/s12887-024-04829-5)

Supplementary Figure 1: Coding tree demonstrating the analytic process

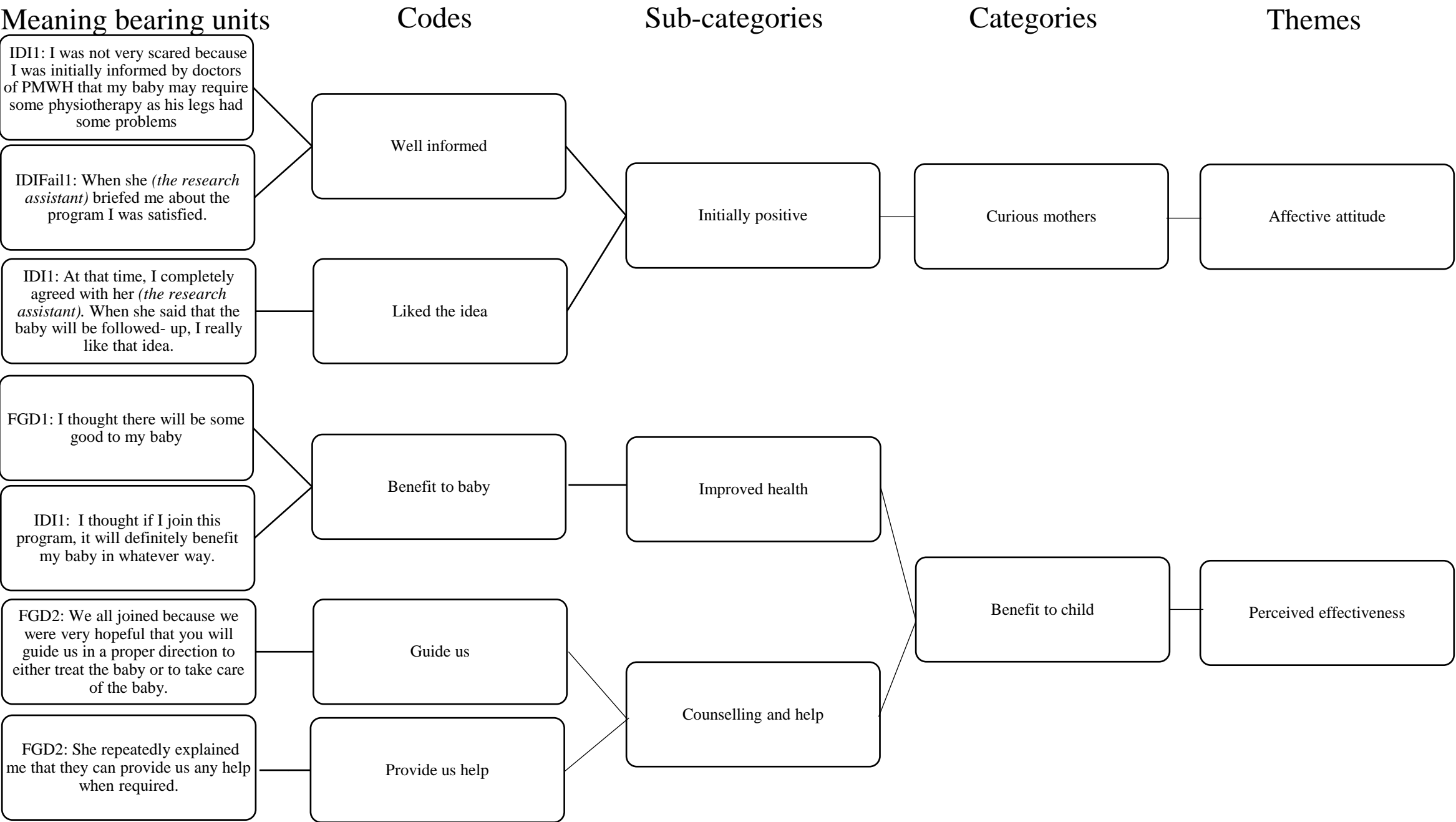

Supplement: Supplementary file 1 — Supplementary Material 1. [file 12887_2024_4829_MOESM1_ESM.pdf]
