# Supplementary material for: ‘We did everything by phone’: a qualitative study of mothers' experience of smartphone-aided screening of cerebral palsy in Kathmandu, Nepal
Source: BMC Pediatr. 2024 May 22;24:357. doi: 10.1186/s12887-024-04829-5 (PMC11110401; doi:10.1186/s12887-024-04829-5)
Supplement: Supplementary file 2 — Supplementary Material 2. [file 12887_2024_4829_MOESM2_ESM.docx]

### Supplementary material: Interview guide for focus group interview on family perspectives about participating in a mobile-phone aided follow-up of a newborn at risk of motor disability

### Overview of the interview guide:

#### Introduction:

- How is your child doing right now?

#### Core Questions:

- What were your **initial thoughts** about when you heard about the follow-up program?

- How did you **feel about being contacted by phone** (at 6 weeks)?

- Please describe your **experience with installing the smart phone app**.

- Describe your **experience in using the smart phone app** to film your child?

- How did you find the **reminder notifications**?

- How did you feel **sending the videos** to the team of experts for evaluation?

- **If it was possible to get the same assessment of movements done during a visit to the hospital**, how would you have felt about that?

#### Final:

- How did you **feel about the results** of the film analysis?

- Describe your **overall perception** of the program after having taken part in it?

- How would you **advice other parents** in similar situation about joining this kind of follow-up in the future?

**This interview is to be done after families have received the feedback about the videos!**

Consent from each interviewee.

Introduction of the interviewer.

You are all parents who have participated in a mobile phone aided follow-up of your children. You were selected for this group interview as we researchers would like to learn how you as parents have experienced the follow-up program of your child. We have just started using the smart phones in the follow-up and would like to use your answers to improve the program. If it’s ok with everyone, we will use a tape recorder to record the discussion for later translation. What is said in the room should be kept in the room. Does anyone have any questions before we start?

### Introductory questions:

First, I’d like to know a bit your children. All your children had some difficulties after the delivery, but could anyone please start by telling **how is your child doing right now?**

*Probes:*

- Does anyone else have a different experience?
  - Is your baby feeding well?
  - Did your baby have any problems after going home from the hospital?
    - If yes, what had happened?
    - *In case problems are identified, the interviewer should offer to discuss them in more detail after the group interview is done.*

### Core questions:

Thank you for your answers.

Let us go forward and discuss your experiences about smart phone follow-up program. First, I’d like you to think back to the time your children were still admitted in the hospital**.** Maybe some of you can remember the time that our research nurse Asmita first told you about this follow-up study.

- **What were your initial thoughts about when you heard about the follow-up program?** *Probes:*
  - Does anybody remember how you felt after discussing with Asmita-mam?
  - What were the reasons that made you join the study?
  - Was there something in particular were you **hoping to gain** from participating in the follow-up study? Something that you feel like could benefit you or your child?
  - Do you feel like you had any other options than joining the study?
    - Please tell us more.

We experienced some delay with preparing the smart phone app used for the follow-up. Some of you might have gone home from the delivery hospital without installing the app. You were later contacted by phone by a research assistant when the smart phone app was ready to be installed. All parents were called when your child was around 6 weeks old to check how your child was doing.

- How did you feel about being contacted by phone*?*
  - - How was the discussion with the research assistant?
- Please describe your experience with installing the smart phone app.
  - *Probes:*
    - How many of you installed the app at the hospital?
    - How many did it at home?
    - Was it easy to follow all the instructions given by the research assistant at the hospital / by phone?
      - Did anyone have any other experience?
      - Please describe any problems you had with the installation

Thank you for your answers. I would now like to move on to discuss your experience with using the app to film your child. Before we do that, **is there anything more you would like to add about the thoughts you had about the follow-up before actually making the films?**

- **Could anyone please start by describing your experience in using the smart phone app to film your child?**

*Probes:*

- Please tell us what exactly you did to film your child?
  - What do others think when hearing this?
  - Did any of you do the filming it differently?
- **What were the challenges you experienced with filming?**

*Probes:*

- - For example, some parents have previously told us that they had difficulties with using the smart phone or finding good internet connection or understanding the instructions written in the app. **Does any of this sound familiar to you?**
    - Please tell me more about that!
- Okay, now we’ve discussed some of the challenges you faced when filming your child. Yet all of you have successfully sent us at least one video and that’s really good, well done! **Could anyone describe some things that made filming your child easier?**

*Probe:*

- - For example, did you receive instructions from research assistant or did some of your family members help you in filming?
  - What helped you to film your child?
- The app used for filming is designed to send in three pop-up notifications related to first occasion of filming followed by two more notifications to remind you of second filming. **Do you remember receiving such notifications?**
  - **How did you find these notifications?**
  - *Probes:*
    - Did you understand the text used in notifications?
  - The idea of the notifications was to remind you parents when it’s time to film your child. Yet, we found that most parents needed to be contacted by a phone call reminding you to send in the films.
    - **Did any of you send in a film by yourself without a phone call reminder?**
      - If yes, what helped you to send in your films by yourself?
      - If no, how did you feel being called and reminded to send in your films?
- After you filmed the videos with the app, the videos were sent to a team of experts for evaluation. **How did you feel sending the videos to this expert team for evaluation?**

*Probe*

- - - Some parents have described us that they felt worried about sending a video of their child while for others it did not seem to be an issue. Could you please describe any worries you might have felt related to filming?
      - Does anyone think otherwise?
    - It took some time to receive the response from the experts. How did you feel about waiting for the response?
- It is currently not possible to do the analysis of your child’s movement directly in the hospital as a team of experts needs to look at the videos. **If it was possible to get the same assessment of movements done during a visit to the hospital, how would you have felt about that?**

*Probes*

- - - Please describe the drawbacks and benefits of filming the child at home and having evaluation done remotely by looking at the films?
    - Would any of you choose to film at home even if the same assessment could be done at a hospital visit?
      - Why / why not?
    - Can you please describe some of the benefits of doing the assessment at the hospital?
    - What about the benefits of doing it at home using a smart phone?

### Final questions:

Thanks again for your answers. We are nearing the end of the interview now.

**Is there anything else related to the filming itself you’d like to tell me before we go further to discuss your current situation?**

Okay then, before we finish, I would like discuss what happened to you and your child after you had sent the films.

- **What kind of feedback did you receive about the results of the film analysis?**

*Probes:*

- - Can anyone please describe what you were told by the research assistant who phoned you?
    - Did anyone receive another type of feedback?
  - How did you feel receiving these film analysis results?
    - Does anyone else have a different experience?
    - Did the results you received somehow change the way you feel about the future of your child?

- - *If the child had abnormal GMA and was referred to SGCP for rehabilitation:*
    - How was the information given to you by the research assistant by phone?
    - Have you already been to our partner organization Self-help group for Cerebral Palsy in Dhapakhel, Lalitpur, to meet their doctors and other staff?
      - How do you feel about the assistance offered by the Self-help group for Cerebral Palsy?
- It’s time for final questions! You have now completed this smart phone aided follow-up study of your child. **What is your overall opinion of the program after having taken part in it?**

*Probes*

- - What did you get out of participating in the program?

**How would you advice other parents in similar situation about joining this kind of follow-up in the future?**

Thank you for your time and answers. Is there anything else you would like to add before we finish the interview?

*The interview should hang around a while to answer to any questions the participants might have about their child or the study.*
